# The Effect of Early Application of Synthetic Peptides 19-2.5 and 19-4LF to Improve Survival and Neurological Outcome in a Mouse Model of Cardiac Arrest and Resuscitation

**DOI:** 10.3390/biomedicines11030855

**Published:** 2023-03-11

**Authors:** Rika Bajorat, Lena Danckert, Florian Ebert, Theresa Bancken, Stefan Bergt, Felix Klawitter, Brigitte Vollmar, Daniel A. Reuter, Tobias Schürholz, Johannes Ehler

**Affiliations:** 1 Department of Anesthesiology, Intensive Care Medicine and Pain Therapy, Rostock University Medical Center, 18057 Rostock, Germany; 2 Department of Anesthesiology and Intensive Care Medicine, MEDICLIN Müritz-Klinikum, 17192 Waren, Germany; 3 Institute of Experimental Surgery, Rostock University Medical Center, 18057 Rostock, Germany; 4 Department of Intensive and Intermediate Care, University Hospital RWTH Aachen, 52074 Aachen, Germany

**Keywords:** cardiac arrest, cardiopulmonary resuscitation, antimicrobial peptide, Pep19-2.5, Pep19-4LF, neurological outcome, neurological tests, brain injury, biomarker, UCH-L1

## Abstract

The synthetic antimicrobial peptides (sAMPs) Pep19-2.5 and Pep19-4LF have been shown in vitro and in vivo to reduce the release of pro-inflammatory cytokines, leading to the suppression of inflammation and immunomodulation. We hypothesized that intervention with Pep19-2.5 and Pep19-4LF immediately after cardiac arrest and resuscitation (CA-CPR) might attenuate immediate systemic inflammation, survival, and long-term outcomes in a standardized mouse model of CA-CPR. Long-term outcomes up to 28 days were assessed between a control group (saline) and two peptide intervention groups. Primarily, survival as well as neurological and cognitive parameters were assessed. In addition, systemic inflammatory molecules and specific biomarkers were analyzed in plasma as well as in brain tissue. Treatment with sAMPs did not provide any short- or long-term benefits for either survival or neurological outcomes, and no significant benefit on inflammation in the CA-CPR animal model. While no difference was found in the plasma analysis of early cytokines between the intervention groups four hours after resuscitation, a significant increase in UCH-L1, a biomarker of neuronal damage and blood–brain barrier rupture, was measured in the Pep19-4LF-treated group. The theoretical benefit of both sAMPs tested here for the treatment of post-cardiac arrest syndrome could not be proven.

## 1. Introduction

Since antimicrobial peptides (AMPs) were identified as part of innate immunity and were able to neutralize invading pathogens, including bacteria, viruses, fungi and parasites, they increasingly gained relevance due to their unique combination of anti-inflammatory, antimicrobial and immunomodulatory capabilities [1,2]. Limulus anti-lipopolysaccharide (LPS) factor peptide-based AMPs were synthesized as a new class of AMPs (synthetic anti-LPS peptides (SALPs)) that represent a highly efficient tool for the neutralization of endotoxin LPS or lipoprotein and for blocking its further immunopathological cascades in vitro and in vivo [3,4]. The synthetic AMPs Pep19-2.5 and Pep19-4LF were shown to reduce the production of pro-inflammatory cytokines in cell culture, as well as their release into plasma in animal models and in clinical studies, resulting in a suppression of inflammation [4,5,6,7]. Pep19-2.5 exerted beneficial effects on liver inflammation in high-fat-diet (HFD)-fed mice, but the mechanism of action remained unclear. Besides the possible interaction of Pep19-2.5 with LPS or lipoprotein (via toll-like receptor 2 (TLR-2)) in HFD-fed mice, serum carnitine levels were restored, and other positive effects on liver metabolism were mediated by reduced CD36 expression secondary to ERK1/2 inhibition [8]. Pep19-4LF was found to reduce organ injury and organ dysfunction in a rat model of hemorrhagic shock without additional affection of LPS or bacteria [7]. Cardiac arrest (CA) leads directly to global hypoxemia and hypoxia, being most critical for the brain within minutes [9]. Persistent hypoxia further contributes to global cerebral ischemia, which induces neuronal damage [10]. Although successful cardiopulmonary resuscitation (CPR) limits the progression of hypoxic organ injury by reperfusion [11], global ischemia followed by reperfusion leads to an excessive systemic inflammatory response called post-cardiac arrest syndrome [11,12,13]. The innate immune system plays an important role in the development and manifestation of ischemia-reperfusion injury, and TLR2 is a key player in this process [14,15,16]. It is characterized by the release of pro-inflammatory mediators, such as cytokines and adhesion molecules; high concentrations of reactive oxygen species; an influx of peripheral immune and inflammatory cells; and the activation of glial cells [17,18,19,20]. Despite intensive research and optimized, guideline-based clinical management, the long-term prognosis of patients who experienced out-of-hospital cardiac arrest (OHCA) is poor. Only approximately eight percent of patients survive the first 30 days after OHCA [21,22,23]. The majority of these patients suffer from severe cognitive deficits and physical disabilities with low potential for rehabilitation [21]. The survival rate with good outcome and neurological function, defined as Category 1 and 2 brain performance (modified Rankin score of 0–3), is only 8.3% [21]. Unfortunately, the majority of patients with successful resuscitation after OHCA still die in hospital from post-cardiac arrest syndrome represented by severe neurological impairment and multiple organ dysfunction [24].

Pep19-2.5 was reported to be a potent anti-inflammatory agent in a murine sepsis model [4,5]. Pep19-4LF was shown to reduce tumor necrosis factor alpha (TNFα) release from human mononuclear cells in vitro and to attenuate organ injury caused by severe hemorrhage and resuscitation in anesthetized rats [7]. In addition, Pep19-4LF also reduced the activation of the NF-kB (nuclear factor ‘kappa-light-chain-enhancer’ of activated B-cells) pathway significantly in different organs, resulting in the reduced formation of the pro-inflammatory cytokines such as interleukin 6 (IL-6) [7]. Furthermore, it significantly attenuated the decrease in blood pressure in hemorrhagic shock [7]. By that, it seemed to improve microvascular perfusion and to reduce organ ischemia. Thus, it was proposed that Pep19-4LF may help to reduce organ injury and inflammation caused by severe hemorrhage and resuscitations in patients with trauma [7].

Given previous reports on the anti-inflammatory value of AMPs, we hypothesized that the early application of peptides after CA and resuscitation in a mouse model is able to mitigate the excessive secondary immune response of the developing post-cardiac arrest syndrome. Thus, our study aimed to investigate the effects of the AMPs Pep19-2.5 and Pep19-4LF on systemic inflammation, survival and long-term neurological outcome in a murine model of cardiac arrest and resuscitation.

## 2. Materials and Methods

### 2.1. Animals

Female wild-type (WT, C57BL/6J, n = 164 in total) 4–5-month-old mice were used with a body weight of approximately 20 g each. Only female mice were used in this resuscitation model, as male mice were shown to regularly develop urinary outflow obstruction and death from post-renal kidney failure between Days 4 and 10 after CA-CPR [25]. Therefore, in all subsequent studies with this model, only female mice were used [16,26,27]. Animals were housed in a temperature-controlled environment (22 °C) under a 12/12 h dark/light cycle with free access to water and food. All procedures were performed according to national and international guidelines on the ethical use of animals (European Communities Council Directive 86/609/EEC). The experimental protocol was approved by the Ethical Committee for Care and Use of Laboratory Animals (local authority: Landesamt für Landwirtschaft, Lebensmittelsicherheit und Fischerei (LALLF) Mecklenburg-Vorpommern, permission number: LALLF M-V/TDS/7221.3-1-068/15). All efforts were made to minimize animal suffering and to reduce the number of animals needed for the experiments.

### 2.2. Study Groups and Experimental Protocol

**Figure 1:**
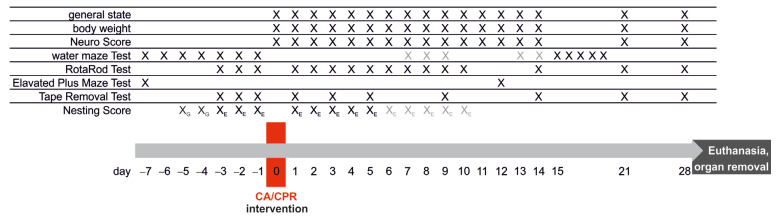
Experimental timeline for neurological and behavioral testing and CA-CPR. black: performed on this day, gray: performed about this day; X_G_: groups of mice, X/X_E_: single mouse.

Three groups of mice were assessed in a long-term study with an observation period of 28 days after CA-CPR. One group was the control group (Ctrl., saline solution), and the other two groups were intervention groups, which received either Pep19-2.5 (Intervention Group 1) or Pep19-4LF (Intervention Group 2) immediately after recovery of spontaneous circulation (ROSC). The experimental timeline of the long-term study, shown in Figure 1, depicts the course of the behavioral and neurological testing in relation to the time of CA-CPR.

The main focus was on survival and neurological outcome; therefore, several standardized neurological, learning and behavioral tests were performed. On Day 0 (Figure 1), just before CA-CPR, the mice were randomly assigned to a group.

A standardized, well-established mouse model of cardiac arrest and resuscitation was used in this study [16,25,26,27]. During the experimental time, the animals had free access to water and food. At the end of the observation period, plasma and various organ samples were collected from the long-term survivors and stored for further analysis. Whole blood was collected in citrate tubes, then centrifuged, and the supernatant was removed and stored deep-frozen. Dissected organs were snap-frozen in liquid nitrogen and then stored at −80 °C.

Furthermore, a short-term study was conducted with the same groups (control, Intervention Group 1: Pep19-2.5 and Intervention Group 2: Pep19-4LF) to collect plasma and organ tissues at an early time point after CA-CPR. The short-term animals were used for blood and organ sampling four hours after CA-CPR and intervention.

Both the neurological and behavioral tests as well as plasma and tissue analyses were performed by study group members who were blinded to the randomization list of the animals.

### 2.3. Anesthesia

All interventions were performed under general anesthesia. Mice were anesthetized by intraperitoneal injection of 12 µg/g ketamine (10%; bela-pharm, Vechta, Germany) and 8 µg/g xylazine hydrochloride (Rompun^®^ 2%; Bayer, Leverkusen, Germany). Animals were then immediately intubated employing a 22-gauge (22G) cannula. Mechanical ventilation was initiated with a fraction of inspired oxygen (FiO_2_) of 0.4, a tidal volume of 10 µL/g and a respiratory rate of 120 breaths per minute (MiniVent Model 845, Hugo Sachs, March, Germany).

### 2.4. Protocol of Cardiac Arrest and Resuscitation

The CA-CPR model was conducted as described previously [16,26,27]. Briefly, the anesthetized and mechanically ventilated (inspiratory oxygen fraction (FiO_2_) 0.21) mice were placed on an auto-regulated heating plate to prevent hypothermia. Body temperature was continuously monitored by a rectal thermocouple probe (Effenberger, Pfeffingen, Germany). Needle probe electrocardiography (ECG; Animal Bio Amp, ADInstruments, Oxford, UK) monitoring was initiated. Blood pressure was measured using a non-invasive blood pressure device (NIBP Controller, ADInstruments, Oxford, UK). Data acquisition was performed digitally (LabChart 5 Pro, ADInstruments, Oxford, UK). A central venous catheter (CVC; PE50, ID 0.28 mm; Portex, Hythe, UK) was inserted into the right jugular vein. CA was induced by injection of 80 µg/g potassium chloride (KCl 7.45%; B. Braun Melsungen AG, Melsungen, Germany), and mechanical ventilation was interrupted upon verification of CA by electrocardiography. Resuscitation was initiated 8 min after CA, and precordial chest compressions were started with a frequency of 450/min using a modified sewing machine as described before [25]. Epinephrine (0.4 µg/g, Adrenalin 1:1000, InfectoPharm GmbH & Co. KG, Heppenheim, Germany) was intravenously injected and mechanical ventilation was resumed (220/min; FiO2 1.0). After two min of CPR, respiratory rate was reduced to 120/min, FiO2 to 0.6 and finally to 0.4 20 min after successful resuscitation. After ROSC, all animals were administered 0.2 mL of tempered isotonic saline (B. Braun Melsungen AG, Melsungen, Germany) intravenously for two hours, and simultaneously, both intervention groups received their specific dose of peptides.

### 2.5. Parameters of Recovery Level and Well-Being of the Animals

Body weight was determined daily until 14 days after CA-CPR and then on Days 21 and 28. In accordance with the guidelines for animal experiments, a loss of body weight greater than 30% from baseline was the decision to withdraw the animal from the experiment by administration of an overdose of i.p. injected ketamine/xylazine.

The nestlet (5 cm square of pressed cotton, ZOONLAB GmbH, Castrop-Rauxel, Germany) was placed in the center of the cage before the dark phase (about 6 p.m.). The next morning (8–10 a.m.), the location of the nest or nestlet was documented, and the nest building was evaluated using an established scoring system for nesting [28]. Scores range from score one, meaning no nest, to score six, representing a perfectly built nest, which means the nest resembles a crater.

### 2.6. Synthetic Peptides

The peptides Pep19-2.5 and Pep19-4LF (Brandenburg Antiinfektiva GmbH, Borstel, Germany) were solved in Aqua (Aqua ad injectabilia, B. Braun Melsungen AG, Melsungen, Germany), and aliquots were stored deep-frozen. For intervention, the peptides were freshly thawed, diluted with tempered isotonic saline to the correct concentration and administered via central venous catheter with 0.1 mL/h over 2 h. Intervention Group 1 received 4 µg of Peptide 19-2.5 [4], and Intervention Group 2 received 7 µg of Peptide 19-4LF [7].

### 2.7. Neurological Assessment

The assessment was performed by trained lab members who were blinded to the group randomization of the animals.

NeuroScore: A modified grading score for mice comprising the following items was used for the standardized neurological assessment: level of consciousness, corneal reflex, respirations, righting reflex, coordination and movement activity [29,30]. At the beginning of the experiments and directly before CA-CPR, all animals underwent NeuroScoring. Animals that did not achieve the maximum score of twelve points were excluded from the experiments. Scoring was performed eight times within the first 24 h after CA-CPR, followed by daily assessment for about 10 days and finally repeated on Days 14, 21 and 28 after CA-CPR.

Rota Rod was performed to assess motor function, balance and coordination [31,32]. Mice were trained to run on the rod starting four days before CA-CPR for three consecutive days with three repetitions. The duration from start on the rod to drop-off was recorded. The maximum time on the rotating cylinder (12.5 revolutions/min) was 300 s. Rota rod tests were accomplished daily for ten days starting 24 hrs after CA-CPR and were repeated on Days 14, 21 and 28.

To compare sensorimotor function and dexterity before and after resuscitation, the tape removal test was performed [33]. The tape removal test was carried out using adhesive tape (3 × 3 mm) to the center of the inside of the front paws (left and right), and the animal was placed in a clear box for the observation period while the time was taken. The following time points were measured: first, the contact time, defined as the time point of the mouse’s reaction to the presence of the adhesive tape on the paw and second, the time point of the removal of the adhesive tape by teeth. Mice were trained with a maximum time of 120 s three consecutive days before CA-CPR (baseline data) and in the testing period on Days 1, 3, 5, 9, 14, 21 and 28 after CA-CPR. All times of 120 s or above were recorded as “120 s” [34].

Water Maze: Spatial learning and memory behavior of the animals was tested using the water maze hidden platform task ([35] adapted to mice: 90 cm maze diameter, 5 × 5 cm platform positioned in one of the four quadrants and four large cues positioned on the edge [16,36]). The tank was round and virtually divided into four parts labeled with the geographical directions, which did not correspond to the exact geographical orientation. Water temperature was kept constant at about 23 °C. The movement of animals in the maze was recorded using tracking software (Ethovision, Noldus, Wageningen, The Netherlands), which was also used to analyze latency to reach the platform and swim distances. After the completion of swimming, the animals were dried with a towel and warmed under a heating lamp. For habituation, all animals were allowed to explore the water maze without a platform on the day before the start of the experiments (seven days before CA-CPR). All animals were trained to find the escaped platform, which was located 0.5 cm below the water surface. To prevent the visibility of the platform, low-fat and lactose-free milk was added to the water. The animal was randomly placed in the maze (one of five different positions) and was allowed to find the escaped platform within 120 s. If the animal failed to reach the platform within 120 s, it was manually placed onto the platform. Once on the platform, the animal was allowed to rest for 20 s in order to orientate. Afterwards, another resting time of 20 s in a cage followed before the next trial was started. The detailed time points of all training days are shown in Figure 1. For the memory test (spatial probe), after surviving resuscitation, all animals had to fulfill physical requirements In order to avoid loss of animals from drowning due to general weakness in a catabolic state [16,26]. Based on individual status of recovery, this test was performed between Days 7 and 14 (minimum–maximum) post-CA-CPR (see Figure 1, light gray crosses). Then, spatial probe swimming was performed two times for 120 s without the platform. For the calculations, the values of the two tests performed (start positions: SW and SE) were both combined and evaluated separately. Afterwards, the training started again with the same protocol but with a different position of the hidden platform in the tank.

### 2.8. Blood Sampling and Tissue Preparation

For organ removal, mice were deeply anesthetized by intraperitoneal injection of ketamine/xylazine (see Section 2.3. Anesthesia above). Blood was collected via retro-orbital puncture, followed by immediate euthanasia via decapitation. The blood samples were centrifuged (1200× *g* for 10 min), and plasma was aliquoted into cryovials and immediately frozen at −20 °C, where it was stored for later analysis.

The brain was dissected immediately after decapitation and transferred to a filter paper in ice-cold buffered saline. The brain was divided into its two hemispheres. The left cerebral hemisphere was rotated onto the cut surface and divided along the longitudinal axis of the cerebrum. Both parts were transferred into tubes and snap-frozen in liquid nitrogen. The tissues were then stored at −80 °C until mRNA isolation. The right hemisphere was directly fixed in 4% formalin (Formafix, Grimm med. Logistik GmbH, Torgelow, Germany) and later stored paraffin-embedded.

### 2.9. Analysis of Plasma Samples

To assess the inflammatory response after CA-CPR and intervention, the cytokines IL-6, interleukin 1β (lL-1β), TNFα and the signal molecule vascular endothelial growth factor A (VEGF-A) were measured in plasma at an early time point (four hours) and after 28 days after CA-CPR. In addition, a neurochemical biomarker for early central ischemic brain injury ubiquitin C-terminal hydrolase-L1 (UCH-L1) was determined in the four-hour plasma samples only [37]. All measurements were performed using electrochemiluminescence-based immunoassays (MESO QuickPlex SQ 120, Meso Scale Discovery (MSD), Rockville, MD, USA). All samples were measured in duplicate with a multiplex U-PLEX assay (analytes: IL-6, lL-1β, TNFα, VEGF-A) and a singleplex assay for the analyte UCH-L1 according to the manufacturer’s recommendations. The readouts were evaluated using the DISCOVERY WORKBENCH^®^ 4.0 software (MSD, Rockville, MD, USA). For the purposes of statistical analyses, any value that was below the lowest limit of detection (LLOD) was considered negative and assigned a value of 0 pg/mL in the assay.

### 2.10. mRNA Expression Analyses from Tissue Samples

The mRNA extraction was performed with the column-based protocols of QIAGEN’s RNeasy Kits (Quiagen GmbH, Hilden Germany) in conjunction with an on-column digestion step with DNAse I. According to the manufacturer’s instructions, the RNeasy Fibrous Tissue Mini Kit was used for the heart and the RNeasy Lipid Tissue Mini Kit was used for the brain except for the hippocampus. For the latter, the RNeasy Micro Kit was used due to its weight (<5 mg). The organs were stored on dry ice during processing, and a maximum of 30 mg of the samples was collected. Tissue homogenization was carried out with the TissueLyser LT (Qiagen, Hilden, Germany) and one stainless steel bead (5 mm). RNA was eluted in 20–50 µL Rnase-free water, and the concentration was determined with BioPhotometer (Eppendorf, Hamburg, Germany).

Two-step RT-qPCR method: All RNA samples were subjected to cDNA synthesis using the SensiFAST™ cDNA Synthesis Kit (Meridian Bioscience, Inc.; Ohio, USA) according to the manufacturer’s recommendations and a total of 500 ng of RNA. No-RT controls were included in any reaction. cDNA samples were kept at −20 °C until further processing.

All primers in the study were designed using the NCBI Primer-BLAST tool [38] or otherwise specified in the supplements (see Appendix A, Table A1: List of qPCR-Primers).

The SensiFAST™ SYBR No-ROX Kit (Meridian Bioscience, Inc.; Newtown, OH, USA) was used for amplification with a constant cDNA concentration of 1.25% in 20 µL total reaction volume according to the manufacturer’s recommendations. All reactions were carried out in duplicates unless otherwise noted and included NTC an. PCR cycling was performed at 95 °C for 2 min, followed by 40 cycles of denaturation at 95 °C for 5 s, annealing at 60 °C for 10 s and extension at 72 °C for 20 s, and melting curve analysis from 60 to 95 °C at a rate of 5 °C/s. For thermal cycling and detection, the qTOWER3G real-time PCR detection system (Analytik Jena AG, Jena, Germany) was used. The standard deviation (SD) of the duplicates was calculated, and samples that exhibited SD > 0.5 were considered inconsistent and were excluded. The best reference genes, ribosomal protein S7 (S7) and peptidylprolyl isomerase A (Ppia), were analyzed in the brain tissue of each biological group by using NormFinder software [39].

### 2.11. Statistics

Results are presented as mean ± standard deviation (SD), mean ± standard error of the mean (SEM) or median (boxplots showing the quartiles, the 5th and 95th percentiles (whiskers), the median (line) and the mean (x)). Survival curves are plotted as Kaplan–Meier and log-rank (Mantel–Cox) tested. Neurological and behavioral tests as well as the plasma and mRNA expression levels were assessed, and significance was tested using the Friedman test for dependent and the Kruskal–Wallis test for independent samples (SPSS 27), followed by pairwise analysis if indicated. The level of significance was set to *p* ≤ 0.05.

## 3. Results

A total of 127 of 164 animals were included in the analysis. For long-term observations, 109 of 127 animals could be evaluated, with 37 animals in the control group (Ctrl.), 35 treated with Pep19-2.5 and 37 treated with Pep19-4LF in the intervention groups (Pep19-2.5, Pep19-4LF). As survival curves began to diverge at approximately four hours after CA-CPR, a short-term animal cohort was formed consisting of the same three groups to implement organ harvesting for biomarker and mRNA expression analyses. In this short-term cohort, 18 of 127 animals were included, with 6 animals per group. Reasons for the exclusion of 37 of the 164 animals were technical or medical complications, such as difficulties with intubation (n = 10), bleeding during central venous line insertion (n = 8), unsuccessful CPR (n = 7), ROSC time greater than 120 s (n = 4) or death before CA (n = 2). In addition, six animals from the short-term cohort did not survive until the time of organ removal after ROSC. In all these cases, the complete datasets of the animals were removed from the analysis.

### 3.1. Long-Term Survival after Eight-Minute Cardiac Arrest and Resuscitation

**Figure 2:**
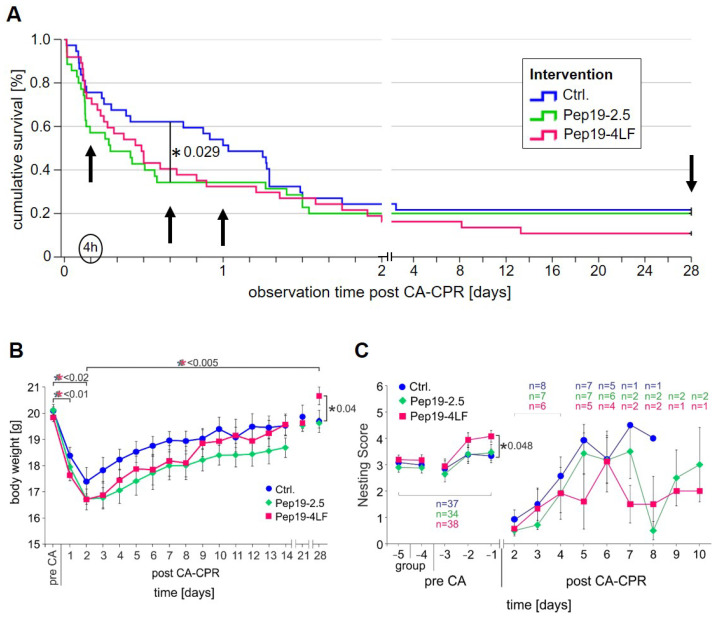
Survival and recovery parameters after CA-CPR. (**A**) Kaplan-Meier plot for mouse survival after successful resuscitation following 8 min of cardiac arrest (CA) within the 28-day observation period. No significant differences between the different groups (controls=Ctrl. (blue): n = 37; Pep19-2.5-treated (green): n = 34; and Pep19-4LF-treated (berry): n = 37) were detected in survival at the end of observation (*p* = 0.465). At 16 h of observation, the difference between the groups was greatest and between control and Pep19-2.5-treated with *p* = 0.029 significant. (**B**) Course of body weight in the observation period of 28 days of all groups was presented as mean ± SEM. Tendentially, the body weight of the control mice does not decrease as much as in the treated groups on Day 2 post-CA-CPR. Thereafter, the animals of all groups gain weight continuously. There was no significant difference between the groups over the time period. Only on Day 28 did the Pep19-4LF treated animals (n = 5) show a higher body weight compared to the control animals (n = 8; *p* = 0.040). (**C**) The nesting behavior (mean ± SEM; n number of animals) was not significantly different between the investigated groups on different days as well as the time (days) to reach the initial nesting score after CA-CPR. The difference between control and Pep19-4LF groups was just not significant on Day 5 (*p* = 0.056). On Days 7 and 8, statistical comparison between groups was not performed because only 1 control animal and 2 animals per peptide treatment group were left in the experiment. For the animals that had reached the nesting score before resuscitation, the experiment ended and so did the single animal housing. Schemes follow the same formatting.

Of the control animals, 8 of 37 (21.6%) were long-term survivors after the 28-day observation period. The survival rate was lower in the intervention groups treated with Pep19-2.5 and Pep19-4LF immediately after ROSC (see Figure 2A). The survival rate after 28 days was 20% (7 out of 35) in the group treated with Pep19-2.5 and 13.5% (5 out of 37) in animals who were administered Pep19-4LF, respectively. Survival was not significantly different between the two intervention groups and controls (*p* = 0.474). Neither the group comparison between controls and the Pep19-2.5 treatment group (*p* = 0.368), nor between controls and Pep19-4LF-treated animals (*p* = 0.217) revealed significant differences after the 28-day observation period.

As visible in Figure 2A, a divergence of the KaplanMeier survival curves was observed four hours after CA-CPR. At this early time point after CA-CPR, controls tended to show a higher survival rate (28 of 37, 75.7%) compared to animals treated with Pep19-2.5 (19 of 35, 54,3%, *p* = 0.084), which was not observed between controls and the Pep19-4LF group (28 of 37, 75,7%, *p* = 0.886). Four hours after CA-CPR, no difference in the survival rate was observed between the two peptide-treated groups (*p* = 0.123). The maximum difference between the survival rates of the controls (23 of 37, 62.2%) and the peptide-treated groups (Pep19-2.5: 12 of 35, 34.3%; Pep19-4LF: 16 of 37, 43,24%) was observed approximately 16 h after CA-CPR. At this time point, the difference between the control group and the Pep19-2.5-treated group reached significance (*p* = 0.029), while the difference between the control group and the Pep19-4LF-treated animals was not significant (*p* = 0.139). From about 32 h after CA-CPR, this trend disappeared.

The values presented in Table 1 provide an overview of the basic clinical parameters before and after CA-CPR in the different intervention groups. There were no differences between the groups, with the exception of lower blood pressure values before CA in controls compared to the intervention groups (see Table 1).

When considered pairwise, blood pressure was significantly higher in Pep19-4LF (*p* = 0.008) compared to controls, which was not present in Pep19-2.5-treated animals (*p* = 0.054). After CA-CPR, the body temperature was kept constant in all groups to avoid influencing hypothermia.

In the present study, none of the resuscitated mice fulfilled the criterion of a loss of body weight of more than 30% from baseline. In all groups, body weight decreased to a minimum on Day 2 after CA-CPR compared to baseline (Ctrl.: 17.4 ± 1.5 g, 86.5% (*p* < 0.0001); Pep19-2.5: 16.7 ± 1.0 g, 83.09% (*p* < 0.0001); Pep19-4LF: 16.7 ± 1.0 g, 84.22% (*p* = 0.014)) and started to increase again during the observation period (Day 28: Ctrl.: 19.7 ± 1.1 g, 98.01%; Pep19-2.5: 19.6 ± 0.5 g, 97.56%; Pep19-4LF: 20.7 ± 0.7 g, 104.19%, Figure 1B). Control mice tended to show a smaller loss of body weight compared to the peptide-treated groups (Figure 2B). At the end of the observation period, the Pep19-4LF-treated mice (20.7 ± 0.7 g; n = 5) had a higher weight compared to the control animals (19.7 ± 1.1 g, *p* = 0.040, n = 8), which was not observed in Pep19-2.5-treated animals.

The same trend was observed for nesting. Nesting behavior did not differ significantly between the studied groups at different time points nor did the time to reach baseline nesting scores again. The surviving animals of the control group nevertheless reached the initial nesting scores two days earlier compared to the peptide groups (see Figure 2C: *p* = 0.276; Ctrl. (n = 8): until Day 8; Pep19-2.5 (n = 7): until Day 10; Pep19-4LF (n = 7): until Day 10).

### 3.2. Neurological Assessment

**Figure 3:**
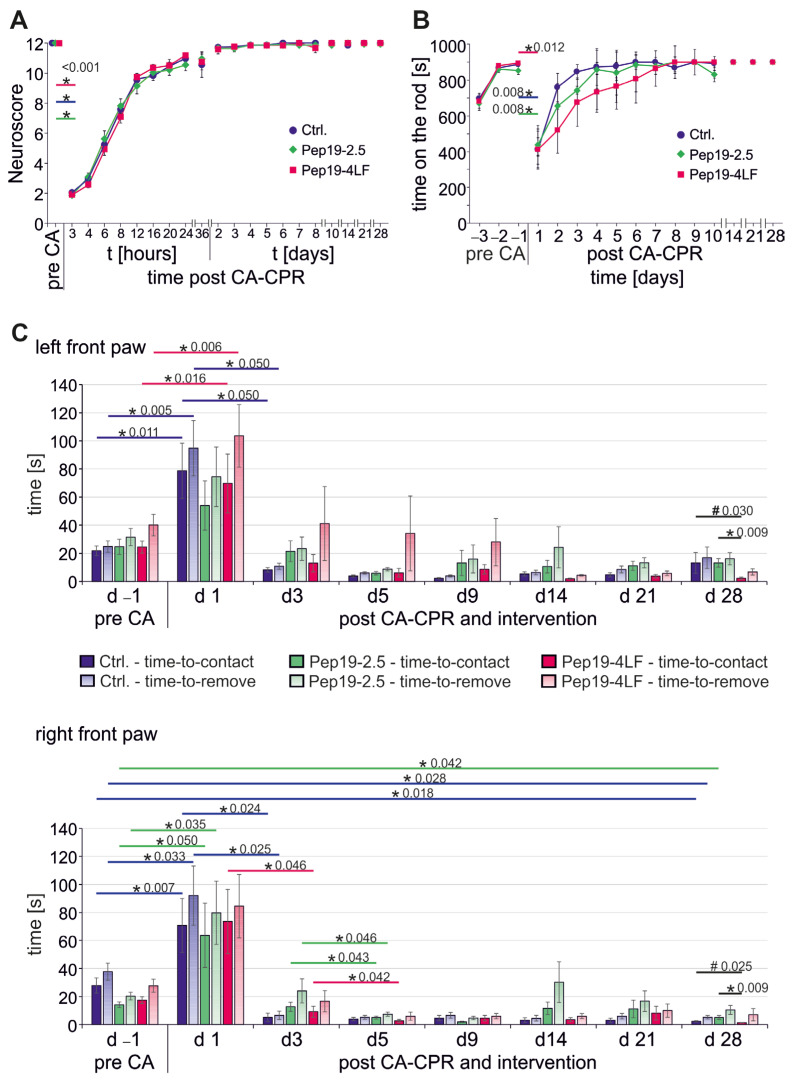
Neurological behavior over a 28-day observation period after CA-CPR. All graphs presented as mean ± SEM. (**A**) The NeuroScore of the animals was almost identical between the intervention groups over the observation period. (**B**) The differences in courses of RotaRod between the intervention groups after CA-CPR over the observation period were not significant (*p* ≥ 0.230). (**C**) In the tape removal test, significant differences between the intervention groups in sensory perception (time-to-contact) were found bilaterally only on Day 28 after CA-CPR (# left: *p* = 0.030; right: *p* = 0.025; further pairwise analysis showed a significant difference between the two peptide-treated groups * left: *p* = 0.009; right: *p* = 0.009).

A NeuroScore of 2 as the lowest level was observed in all three groups three hours after CA-CPR, and the baseline value was almost regained on the second day after CA-CPR. The progress of the NeuroScore levels was identical between the three groups within the observation period (Figure 3A).

Similar results were obtained from the RotaRod test (Figure 3B). All tested animals, regardless of their group affiliation, showed a significant decrease in their walking performance on the rod one day after CA-CPR (Day –1 versus (vs.) Day 1: Ctrl.: 888 ± 45 s vs. 411 ± 409 s, *p* = 0.008; Pep19-2.5: 855 ± 130 s vs. 438 ± 342 s, *p* = 0.008; Pep19-4LF: 897 ± 18 s vs. 413 ± 356 s, *p* = 0.012, Figure 2B). Animals from the control group tended to reach the baseline performance on the RotaRod again more easily, but the differences were neither significant compared to the intervention groups nor between the intervention groups themselves (*p* ≥ 0.230).

In the tape removal test, there were bilateral significant differences between the intervention groups in sensory perception (time-to-contact) on Day 28 after CA-CPR (Figure 3C, black #: left: *p* = 0.030; right: *p* = 0.025), with a higher sensory perception in the group treated with Pep19-4LF. Subsequent pairwise analysis showed that the significant difference was between the two peptide-treated groups (Figure 3C, black *: left: Pep19-2.5 (13.14 ± 3.0 s) vs. Pep19-4LF (2.3 ± 0.8 s): *p* = 0.009; right: Pep19-2.5 (5.00 ± 1.4 s) vs. Pep19-4-LF (1.25 ± 0.2 s): *p* = 0.009).

By comparing the left and right sides, the control mice perceived the tape significantly faster on the right side on Day 28 after CA-CPR (time-to-contact: *p* = 0.027). This difference did not remain valid for the removal of the tape on Day 28 after CA-CPR (*p* = 0.090). The day before resuscitation, the mice in the control group interestingly tended to be faster on the left side (see Figure 3C; time-to-remove d –1: *p* = 0.054). Besides this, no obvious lateral differences between the right and left paws were observed in the peptide-treated groups.

**Figure 4:**
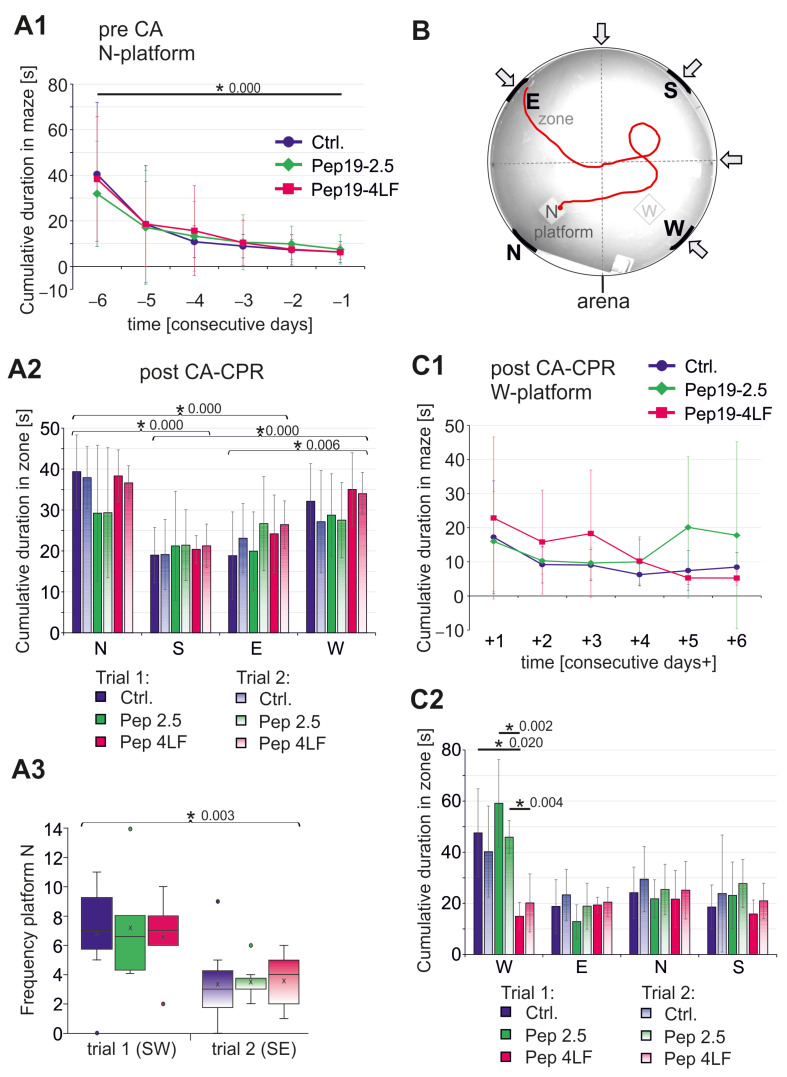
Testing of spatial learning and memory using the water maze task. (1, 2) given as mean ± SD and (3) were presented as boxplots showing the quartiles, the 5th and 95th percentiles (whiskers), the median (line) and the mean (x). (**A1**) Cumulative duration in maze during the learning phase pre-CA using platform at N position. In all groups, the mice have learned where the invisible platform was placed (d –6 vs. d –1: *p* = 0.000). (**A2–3**) Spatial probe post-CA-CPR without N-platform. (**A2**) The cumulative duration in the individual zones of the arena without N-platform (first and second trials) was not different between zone N vs. W and zone S vs. E. But all other comparisons of cumulative durations in the zones showed significant differences (*p*-values see A2). (**A3**) Frequency at which the position of the platform was directly swum through during a 2 min time in the water maze. Significance tested between Trial 1 and Trial 2 (*p* = 0.003). (**B**) Sample photo of mouse tracking (red line) on learning day 3 in the arena showing zones (N, S, E, W), position of invisible platform (N) and different starting positions (arrows) used in alternating. (**C1**) Cumulative duration in maze during the learning phase post-CA-CPR using position of Platform W. (**C2**) Spatial probe post-CA-CPR without W-platform. Cumulative duration in the individual zones of the arena without W-platform.

All animals were also tested for their spatial learning and memory behavior using the water maze task. Before CA-CPR, all animals underwent a learning phase with the invisible platform in the N position over six consecutive days with alternating starting points (Figure 3B). Independently from their group, the animals learned to find the hidden platform position quickly on Day 0 (* Figure 4A1; Ctrl. (n = 37): *p* < 0.0001; Pep19-2.5 (n = 35): *p* = 0.002; Pep19-4LF (n = 37): *p* < 0.0001; Figure 4B).

After CA-CPR, animals were tested for memory regarding the position of the invisible platform. No difference was found between the three study groups in terms of memory, represented by the time the animals spent in the quarter where platform was located during the learning phase (1st trial (start SW), *p* = 0.957; 2nd trial (start SE), *p* = 0.878, see Figure 4A2). Comparing the durations of stay in each zone of the pool using Friedman’s two-factor analysis of variance for ranks revealed that the mice clearly stayed significantly more often in Zone N compared to Zones S and E (*p* ≤ 0.0001), while the comparison to the W zone just missed the significance level (*p* = 0.051, see Figure 4A2). Compared to the W zone, it was also evident that the mice spent significantly less time in the S zone (*p* < 0.0001) and E zone (*p* = 0.006, see Figure 4A2). However, there was a significant difference in the frequency of the mice to reach the exact position of the platform between the first and second trials, which were performed immediately after each other (*p* = 0.003; Figure 4A3).

After the memory test, re-learning was examined after surviving CA with an invisible platform in the W zone. The results show a learning effect (Figure 4C1), although not as obvious as during the first learning phase (Figure 4A1). The time taken to find the invisible W-platform on the first day of the learning phase (Figure 4C1: d + 1) was significantly shorter than it had been for the N-platform (Figure 4A1: d –6; Wilcoxon test: *p* = 0.033), but there were no differences regarding the study groups. Animals treated with Pep19-4LF after CA-CPR spent approximately equal times in each zone (red, Figure 4C2). In comparison, the mice of the control and Pep19-2.5 groups stayed slightly longer in the W zone, but the difference from the times spent in the other zones (E, N, S) was not significant (Figure 4C2). However, the average swimming time in the W zone was significantly lower in the Pep19-4LF-treated group than in the control group and of the group that had received Pep19-2.5 (Trial 1-starting point SE: *p* = 0.006; Ctrl. vs. Pep19-4LF: *p* = 0.020; Pep19-2.5 vs. Pep19-4LF: *p* = 0.002; Trial 2-starting point NE: *p* = 0.015; Ctrl. vs. Pep19-4LF: *p* = 0.066; Pep19-2.5 vs. Pep19-4LF: *p* = 0.004, see Figure 4C2). The frequency at which the position of the W-platform was directly swum through during a 2 min time in the water maze was not different between Trial 1 (SE) and Trial 2 (NE).

### 3.3. Plasma Biomarker Levels

**Figure 5:**
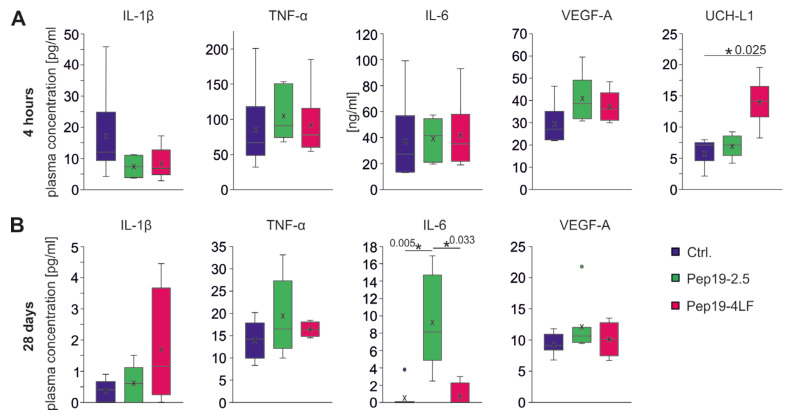
Plasma levels of IL-1β, TNF-α, IL-6, VEGF-A and UCH-L1 four hours (**A**) and 28 days (**B**) after CA-CPR. Measurements are shown in pg/mL (except IL-6 after 4 h in ng/mL). Plasma levels were assessed in each group and presented as boxplots showing the quartiles, the 5th and 95th percentiles (whiskers), the median (line) and the mean (x). (**A**) Four hours after CA-CPR, plasma levels of cytokines and VEGF-A were measured at an elevated level compared with 28 days after CA-CPR (**B**). Differences in the comparison of the groups (each n = 6, unless otherwise specified) could not be detected with regard to cytokines (IL-1β: *p* = 0.109; TNF-α: *p* = 0.423; IL-6: *p* = 0.519) and VEGF-A (*p* = 0.076). UCH-L1 levels in the Pep19-4LF treated group (n = 5) were significantly higher than in the control group (n = 4, *p* = 0.025). (**B**) All plasma levels of cytokines and VEGF-A 28 days after CA-CPR were normally low. For IL-6, the group of mice treated with peptide Pep19-2.5 showed significantly higher concentration after CA-CPR (*p* = 0.003; pairwise: Ctrl. (n = 8) vs. Pep19-2.5 (n = 7), *p* = 0.005; Pep19-4LF (n = 4) vs. Pep19-2.5: *p* = 0.033; Ctrl. vs. Pep19-4LF: *p* = 0.864).

The cytokines IL-1ß, TNF-α, IL-6, VEGF-A (hypoxia-induced signaling molecule) and UCH-L1 (biomarker for early ischemic brain injury) were measured in plasma. Plasma levels of cytokines were significantly higher four hours after CA-CPR (Figure 5A) compared to long-term survivors after 28 days (Figure 5B), where levels returned to baseline levels. Among long-term survivors, there were no differences between the intervention groups for the determined cytokines, except plasma IL-6 levels in the Pep19-2.5-treated group were significantly higher compared to the other groups (*p* = 0.003, Ctrl. vs. Pep19-2.5: *p* = 0.005; Pep19-4LF vs. Pep19-2.5: *p* = 0.033, see Figure 5B).

During the early phase after CA-CPR (four hours), cytokine levels were all significantly elevated, but no significant differences were found between the intervention groups (each group n = 6; Figure 5A). The control group showed a trend towards higher IL1-β levels compared to the peptide-treated groups (*p* = 0.109). Plasma VEGF-A concentration (four h mean concentration in pg/mL: each group n = 6, Ctrl. = 29.4, Pep19-2.5 = 40.9, Pep19-4LF = 37.4, see Figure 5A) was increased 3–4-fold compared in long-term survivors (28 d mean concentration in pg/mL: Ctrl. (n = 8) = 9.3, Pep19-2.5 (n = 7) = 12.1, Pep19-4LF (n = 4) = 10.1, see Figure 5B). After four hours, there was a tendency towards higher levels of VEGF-A in the two peptide-treated groups compared to the control group (*p* = 0.076; Figure 5A). In plasma samples from untreated mice lacking CA-CPR, UCH-L1 showed a value of 3.62 ± 1.29 pg/mL (n = 4, not shown in figure), and in the resuscitated groups, their values were at least twice as high after four hours (Ctrl. (n = 4): 5.72 ± 2.5 pg/mL, Pep19-2.5 (n = 5): 6.90 ± 1.97 pg/mL, Pep19-4LF (n = 5): 14.00 ± 4.09 pg/mL; Figure 5A). UCH-L1 levels (Figure 4A) in the Pep19-4LF-treated group were significantly higher than in the control group (*p* = 0.025), but the difference compared to the Pep19-2.5-treated group was not significant (*p* = 0.088). When comparing all three groups, *p* did not reach the significance level (*p* = 0.062).

### 3.4. mRNA Expression Levels Four Hours after CA-CPR in Brain Tissue

**Figure 6:**
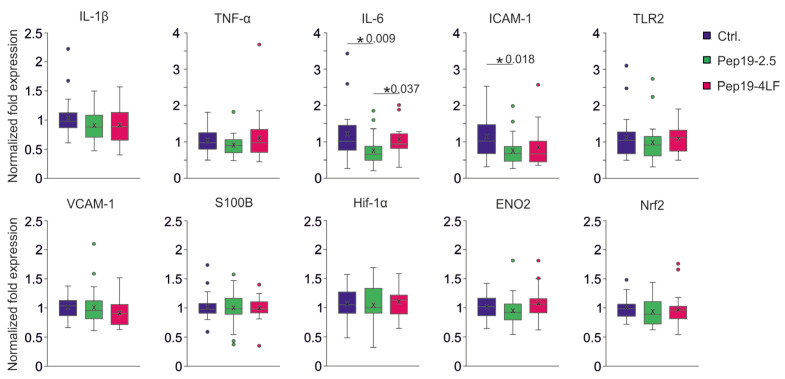
mRNA Expression levels of brain tissue from 4 h after CA-CPR. All results were presented as boxplots showing the quartiles, the 5th and 95th percentiles (whiskers), the median (line) and the mean (x). Normalized expression of each group (n = 6) of cytokines (TNF-α, IL-6, IL1β), cell adhesion molecules (ICAM-1, VCAM-1), TLR2, transcription factors (HIF-1α; Nrf2), neuron-specific marker (S100B, ENO2 (NSE)). All expression differences were Kruskal-Wallis tested. IL-6 mRNA expression level was lower in the Pep19-2.5 compared to the control (*p* = 0.009) as well as the Pep19-4LF group (*p* = 0.037). The level of ICAM-1 mRNA expression was lower in the Pep19-2.5 than in the control group (*p* = 0.018).

In the group receiving Peptide-19-2.5 after CA-CPR, the level of cytokine IL-6 was significantly lower compared to the other groups (Ctrl.: *p* = 0.009, Pep19-4LF: *p* = 0.037, see Figure 6). Increased expression for ICAM-1 was determined in the control group compared to the Pep19-2.5-treated group (*p* = 0.018), while the difference with the Pep19-4LF-treated group did not reach the significance level (*p* = 0.052, see Figure 6).

All expression levels for various immune molecules (IL1β, TNF-α), neuronal markers (S100 calcium-binding protein B (S100B), neuron-specific enolase (ENO2 = NSE, an enzyme involved in cerebral glycolytic energy metabolism)), different signaling molecules (intercellular adhesion molecule 1 (ICAM-1), vascular cell adhesion molecule 1 (VCAM-1)), receptor (TLR2, membrane protein of innate immune system on leukocytes), as well as transcription factors (hypoxia-inducible transcription factor (HIF-1α), nuclear factor erythroid 2-related factor 2 (Nrf2)), were equal in brain tissue at the four-hour time point in the group comparisons (see Figure 6). It should provide a concise and precise description of the experimental results, their interpretation, as well as the experimental conclusions that can be drawn.

## 4. Discussion

To the best of our knowledge, this data set represents the first characterization of the early pharmacological effects of the two different AMPs Pep19-2.5 and Pep19-4LF on post-cardiac arrest syndrome in a mouse model. Animals were assessed in a comparative, randomized, blinded study in intervention groups and a control group. Global indicators, survival and neurological outcome were not improved by treatment with peptides after CA-CPR. Here, the animals did not benefit from the anti-inflammatory effects of the two tested AMPs, which have been demonstrated in several experimental studies in vitro and in vivo [4,5,6,7,40]. This is in contrast to reports from animals after successful resuscitation [41]. In all these studies, an excessive response of the innate immune system as well as the endothelium [40] was observed as a second hit. Consecutively, this led to multi-organ dysfunction, including brain, lung and renal injury. In the present study, long-term outcomes up to 28 days after CA-CPR were assessed using global circulatory, systemic inflammatory and neurological parameters. Interestingly, we did not observe significant differences with a look at the survival curves (Figure 2A) of the three study groups during the 28-day observation period. Animals treated with Pep19-4LF even tended to have a worse outcome compared to controls and to animals treated with Pep19-2.5. A closer look at the survival curve reveals that the first deviation occurred shortly before four hours after CA-CPR, approximately one hour after administration of the peptide or saline solution was terminated. We speculate that this was an attenuating effect of the peptides on the immune response following ischemia–reperfusion caused by the prolonged administration of the peptides. Similar effects were previously described during the administration of the peptide Pep19-2.5 over 24 h in a sepsis mouse model [4,42]. In our study, we decided to use a two-hour administration of AMPs because of the severity of the resuscitation model. For such acute models, the avoidance of any additional stress is fundamental. The longer administration period is associated with risks, such as an additional infection confounding the study results, restriction of animal movement affecting welfare and recovery, and unintentional tearing of the CVC.

Neurologic outcome is of particular interest to the resuscitation model because of the ultimate importance of outcome to survivors. Previous studies using this resuscitation model demonstrated differences after resuscitation in the development of the NeuroScore (general neurological evaluation), the RotaRod test (motor function, balance and coordination) and the water maze test values (spatial learning and memory behavior) underlining their suitability [16,26]. Therefore, the chosen tests are clearly suitable for the detection of potential differences. In this study, the behavioral and learning tests did not show a difference between the three groups. Hence, it can be concluded that the early application of Pep19-2.5 and Pep19-4LF is not able to improve the neurological outcome after CA-CPR. Nevertheless, there is an interesting aspect regarding the tape removal test. A difference In the perception of the sticky tape (time-to-contact, see Figure 3) could be observed on Day 28 after CA-CPR. Animals treated with Pep19-4LF had a higher sensory perception of the tape on both paws compared to the other groups. In a rat study, the tape removal test was appropriate for the detection of brain injury three and seven days after ROSC [34]. In accordance with our results, Albertsmeier et al. showed that a time point one day after resuscitation might be too early for results derived from the tape removal test. Resuscitated rats showed a partial recovery in the neurological deficit score between 24 and 48 h and a final moderate disability after 48 and 72 h, which is comparable to the NeuroScore in combination with the RotaRod that we used in the present study [43]. The learning of the changed position of the invisible platform was lower on Day 1 than in the learning experiment before CA. Since the water maze was not new in the second learning, orientation in the water basin might have been easier for the animals.

Insight into the systemic inflammatory response of mice was obtained by cytokine level in plasma and gene expression analysis from brain tissue. In this regard, two time events were selected by survival curves: long term after Day 28 and short term 4 h after CA-CPR. Plasma levels of cytokines were markedly increased after four hours and decreased significantly until Day 28. Among other effects, in rats with hemorrhagic shock, four hours of continuous administration of Pep19-4LF was shown to attenuate the increase in IL-6 serum concentrations to sham levels [7]. In the present study, no differences in IL-6, TNF-α and IL1-β levels were observed between the intervention groups four hours after CA-CPR. IL-1β and TNF-α are two of the best-characterized early-response cytokines and are often expressed concurrently [11]. These cytokines have a strong pro-inflammatory effect, are secreted by various components of the immune system and central nervous system (CNS [11]), and appear to exacerbate brain damage [44,45]. Our data do not support any significant effects of Pep19-2.5 and Pep19-4LF on these cytokines.

The production of VEGF-A is stimulated by hypoxemia [46]. As a result, endothelial cells produce hypoxia-induced factors that lead to the release of VEGF and ultimately to angiogenesis, as has been elucidated in cell lines [47,48]. In the present study, VEGF-A plasma concentration tended to be higher in the peptide-treated groups than in the control group. However, the difference did not reach the significance level at this early time point. Nevertheless, this suggests that the peptides do not attenuate the increase in VEGF-A after CA-induced hypoxemia.

Importantly, we demonstrated that UCH-L1 was detectable in plasma at a very early stage of global ischemic injury in a mouse CA-CPR model. It has already been demonstrated in animal studies on TBI that UCH-L1 can indicate neuronal damage very early after damaging events [49,50]. Group statistics tended to show an increase in UCH-L1 in the Pep19-4LF-treated group compared with Peptide 19-2.5 and controls, and pairwise comparison even showed a significantly higher plasma level of UCH-L1 in Pep19-4LF-treated mice versus controls. One could speculate that this indicates only the beginning of the increase in UCH-L1 in plasma due to the early time point that was used for the measurement in the present study. Future studies are encouraged to evaluate the potential of UCHL-1 during the further course after CA-CPR. At 24 h after resuscitation, the serum UCH-L1 level in male rabbits was increased, but not in the sham group [51]. However, 48 and 72 h after resuscitation, plasma UCH-L1 levels gradually decreased in the resuscitated groups [51]. It is possible that UCH-L1 is only useful as a biomarker in the first 48 h after CA-CPR. A previous study of UCH-L1 in models of both traumatic brain injury and ischemic stroke in rats indicates that UCH-L1 levels are elevated early and appear to be dependent on the severity of injury [37]. In the present study, we measured UCH-L1 only at one time point (four hours after CA-CPR in the short-term animals) in the acute phase after resuscitation; therefore, we could not correlate UCH-L1 levels with the severity of brain injury. As UCH-L1 is a 24-kDa protein with no known active transport mechanism, it is likely that a breakdown of the blood–brain barrier (BBB) following brain trauma or ischemia is responsible for its release into the circulating blood [37]. Therefore, UCH-L1 might also represent an indirect marker for BBB damage. A recent clinical study correlated UCH-L1, glial fibrillary acidic protein (GFAP) and neuron-specific enolase (NSE) serum protein levels at 24, 48 and 72 h after resuscitation with performance category at 6-month follow-up. UCH-L1 and the combination of GFAP + UCH-L1 values were significantly more correlated with neurological outcome than NSE at 24 h but not at 48 and 72 h [52]. At later time points, only the combination of GFAP + UCH-L1 levels provided similar results to NSE measurements. Across all time points of measurement, the model NSE combined with GFAP + UCH-L1, together with clinical and neurological bedside information, resulted in a significantly more complete diagnostic image [52].

Different mRNA expression levels were examined in brain samples four hours after CA-CPR. The expression of IL-6 was significantly different between the three groups, namely, the Pep19-2.5-treated animals showed a lower expression level compared to both controls and Pep19-4LF-treated animals. This difference could not be measured in the plasma level of IL-6. The fact that at the time of measurement, the peptide-treated groups showed a lower expression level of IL-6 compared to controls did not seem to have an impact on survival, especially not even at this early time point. IL-6 is a pleiotropic cytokine and a crucial pre-inflammatory factor with a central role in host defense and acute inflammatory responses, exhibiting both pro-inflammatory and anti-inflammatory activities [53,54]. In the present study, only another significant difference was found for the expression of ICAM-1. ICAM-1 expression was lower after peptide treatment than in controls and reached significance for Pep19-2.5 compared to controls. In the CNS, ICAM-1 is expressed in microglial cells and astrocytes and in endothelial cells in the white and gray matter. It regulates endothelial and epithelial barrier function and is an important early marker of immune response [55]. An elevated expression of ICAM-1 was also confirmed within hours after the onset of ischemic stroke [56,57], at 6 and 72 h after hemorrhage [58] and in examined hippocampal CA1 regions [43]. In a mouse model of sepsis, an association between BBB disruption and ICAM-1 induction was demonstrated [59]. All other expression analyses for various proteins, transcription factors or receptors are relevant in the context of damage caused by hypoxia (Hif1α, Nrf2, Mif-1), ischemia–reperfusion (Hif1α, Nrf2, Mif-1, TLR2) and inflammation (IL-1ß, TNF-α, IL-6, ICAM-1), particularly in the brain (ENO2) and endothelium (ICAM-1, VCAM-1). However, for TLR2, there is evidence that its expression levels are increased in ischemic tissue [60]. At the 4 h measurement time point, at least, no effect on expression was detected by administration of the anti-inflammatory peptides.

The administration of the peptides was supposed to attenuate the inflammatory response, but this could not be observed. Nevertheless, it is important to keep in mind that only limited insight into gene expression and cytokine levels in blood is provided, as there is only one time point for short- and long-term determination. The missing positive effect of the early peptide application on the other hand needs discussion. One reason for this might be the relatively short time window of peptide application in our study (for 2 h after ROSC) compared to others. Schuerholz and colleagues applied Peptide 19-2.5 over 24 h in a model of sepsis [4], and Yamada and colleagues administered Peptide 19-4LF over four hours after hemorrhage and resuscitation in rats [7]. The early time of sampling for the plasma and expression analyses resulted from the divergence of the survival curves after four hours. Therefore, we could not correlate the biomarker findings with neurological and global outcome parameters, as the animals had to be euthanized for tissue sampling. More values at the time points 12, 24 and 72 h after CA-CPR would be desirable for the longitudinal assessment of immunologic and neurological functions. However, due to the long-term character of the present study with a focus on the intervention with the peptides, this longitudinal approach was not indicated with regard to animal welfare.

On the other hand, key strengths of the present study are the use of a well-established mouse model of CA-CPR, the long duration of observation to assess the long-term outcomes, a very comprehensive protocol for the collection of neurological and cognitive outcome parameters, the blinded and randomized conduction of the study, and blinded analyses of plasma and tissue samples.

## 5. Conclusions

In the present mouse model of cardiac arrest and cardiopulmonary resuscitation, the immediate treatment with synthetic antimicrobial and anti-inflammatory peptides Pep19-2.5 and Pep19-4LF did not provide benefits for long-term survival or neurological outcome. This was confirmed by evidence of no significant differences between the study groups regarding early-phase inflammatory mediators as well as neuronal and endothelial injury markers four hours after cardiac arrest and resuscitation. Modulations in dose, time of application and duration of administration might lead to different results. We were able to show that increased levels of neuronal injury markers such as UCHL-1 can be measured during the early phase after resuscitation, which might have potential for future longitudinal studies on neurological outcome, especially in combination with other markers like GFAP and NSE.

## Figures and Tables

**Table 1:** Global parameters of CA-CPR. The significance levels were calculated using the Kruskal–Wallis test.

	Experimental Groups			
Intervention:	Ctrl. (NaCl 0,9%)	Pep19-2.5	Pep19-4LF	
	n = 37	n = 34	n = 37	
Parameter	Mean ± SD	Mean ± SD	Mean ± SD	*p* Value
**Baseline before CA**				
heart rate (1/min) MAP (mm Hg) body temperature (°C)	210.78 ± 23.81	220.71 ± 23.52	220.34 ± 25.15	0.141
	64.95 ± 8.26	68.79 ± 9.03	70.32 ± 7.67	0.021 *
	36.21 ± 0.28	36.06 ± 0.20	36.10 ± 0.20	0.033 ^†^
**CA**				
ROSC time (s) dosage epinephrine (μg) extubation (min)	63.32 ± 27.74	70.76 ± 37.43	64.39 ± 43.85	0.375
	13.38 ± 3.14	14.04 ± 3.84	12.89 ± 3.61	0.276
	169.53 ± 17.81	171.31 ± 14.22	175.06 ± 17.86	0.376
**1 h after CA**				
heart rate (1/min)	328.56 ± 75.80	352.00 ± 88.82	337.91 ± 78.92	0.763
MAP (mm Hg)	57.33 ± 8.98	56.00 ± 4.86	52.67 ± 2.92	0.592
body temperature (°C)	36.25 ± 0.26	36.30 ± 0.35	36.33 ± 0.27	0.379
**2 h after CA**				
heart rate (1/min)	273.57 ± 71.99	298.64 ± 75.87	300.11 ± 76.86	0.448
MAP (mm Hg)	57.80 ± 7.52	55.00 ± 0.00	53.00 ± 2.00	0.664
body temperature (°C)	36.36 ± 0.36	36.39 ± 0.34	36.44 ± 0.31	0.356

* In controls, blood pressure before the induced cardiac arrest was lower than in peptide-treated groups. When considered pairwise, blood pressure was significantly higher in Pep19-4LF (*p* = 0.008), and in Pep19-2.5, the difference did not reach significance (*p* = 0.054). ^†^ Before cardiac arrest induction, body temperature was lower in Pep19-2.5-treated animals, but when considered pairwise, the difference was significant only compared to control animals (*p* = 0.010), but not compared to Pep19-4LF (*p* = 0.099).

## Data Availability

The authors confirm that the data supporting the findings of this study are available within the article and its Appendix. Raw data were generated at Rostock University Medical Center, Department of Anesthesiology and Intensive Care Medicine using the animal core facility at the Institute of Experimental Surgery. Derived data supporting the findings of this study are available from the corresponding author R.B. on request.

## Appendix A

**Table A1:** List of qPCR-Primers. The primers used for the normalization are highlighted in gray.

Gene	Gene Name	Primer	Sequence	Primer Length [bp]	Product Length [bp]	Efficiency [%]	Source	Primer Design	Primer Concen-tration	R^2^
***Hif1a***	Hypoxia inducible factor 1 subunit alpha	Forward	TCAAGCAGCAGGAATTGGAAC	21	181	95	NCBI Primer- BLAST	Exon junction	400 nM	0.99
		Reverse	CTCATCCATTGACTGCCCCA	20						
***s100b***	S100 calcium binding protein B	Forward	GGTGACAAGCACAAGCTGAAG	21	91	104	NCBI Primer- BLAST	Exon junction	400 nM	0.97
		Reverse	CTTCCTGCTCCTTGATTTCCTCCA	24						
***eno2***	Enolase 2	Forward	AGGTGGATCTCTATACTGCCAAA	23	98	96	PrimerBank ID14290500a1	Intron Inclusion	200 nM	0.99
		Reverse	GTCCCCATCCCTTAGTTCCAG	21						
***ICAM-1***	Intercellular adhesion	Forward	GTGGGTCGAAGGTGGTTCTT	20	188	105	NCBI Primer- BLAST	Exon junction	400 nM	1
	molecule 1	Reverse	CCGAGGACCATACAGCACG	19						
***VCAM-1***	Vascular cell adhesion molecule 1	Forward	CAAAAAGGGACGATTCCGGC	20	187	123	NCBI Primer- BLAST	Intron Inclusion	400 nM	0.96
		Reverse	GTTTCGGGCACATTTCCACA	20						
***Nrf2***	Nuclear factor, erythroid 2 like 2	Forward	AGCAGGACATGGAGCAAGTT	20	96	117	NCBI Primer- BLAST	Intron Inclusion	400 nM	0.98
		Reverse	CAGCGGTAGTATCAGCCAGC	20						
***Tnf***	Tumor necrosis factor	Forward	CCTGTAGCCCACGTCGTAG	19	148	120	PrimerBank ID133892368c3	Intron Inclusion	500 nM	0.98
		Reverse	GGGAGTAGACAAGGTACAACCC	22						
***Il1*β**	Interleukin 1 beta	Forward	ATGAAAGACGGCACACCCAC	20	175	129	NCBI Primer- BLAST	Intron Inclusion	400 nM	0.99
		Reverse	GCTTGTGCTCTGCTTGTGAG	20						
***Il6***	Interleukin 6	Forward	GAGGATACCACTCCCAACAGACC	23	141	106	Aachen 078	Intron Inclusion	400 nM	0.99
		Reverse	AAGTGCATCATCGTTGTTCATACA	24						
***Tlr2***	Toll like receptor 2	Forward	ACCTGAGAATGATGTGGGCG	20	202	103	NCBI Primer- BLAST	Non-Exon-junction	400 nM	0.99
		Reverse	CATTTGCCCGGAACGAAGTC	20						
***Ppia***	Peptidylprolyl isomerase A	Forward	GGCAAATGCTGGACCAAAC	19	110	98	Sundaram et al., PLoS one 2019.	Intron Inclusion	400 nM	0.99
		Reverse	CATTCCTGGACCCAAAACG	19						
***s7***	Ribosomal protein S7	Forward	AAAGTTCAGTGGCAAGCACG	20	99	99	NCBI Primer- BLAST	Intron Inclusion	400 nM	0.99
		Reverse	CTGGGGCGCTTCTGCTTATT	20						
